# Brain asymmetry in cortical thickness is correlated with cognitive function

**DOI:** 10.3389/fnhum.2014.00877

**Published:** 2014-10-29

**Authors:** Chong Chen, Yuki Omiya

**Affiliations:** ^1^Department of Psychiatry, Hokkaido University Graduate School of MedicineSapporo, Japan; ^2^Department of Anatomy, Hokkaido University Graduate School of MedicineSapporo, Japan

**Keywords:** structure-function relationship, brain asymmetry, cortical thickness, aging, wisdom, gender difference, working memory, cognitive function

Man is, of all the animals, the one whose brain in the normal state is the most asymmetrical. He is also the one who possesses most acquired faculties. Among these faculties… the faculty of articulate language holds pride of place. It is this that distinguishes us the most clearly from the animal.

—Broca (1877)

A brain is considered to be asymmetrical if the two hemispheres are different from each other, structurally, and/or functionally. The most well established brain asymmetry is related to language, in which the specialized left hemisphere is markedly expanded in most people, especially in Broca's speech area, the planum temporale (Toga and Thompson, 2003). Brain asymmetry is believed to be evolutionally adaptive, since unilateral computation and control might be more efficient than bilateral. It also reduces possible interference between hemispheres, freeing up the opposite hemisphere for other specialized functions (Toga and Thompson, 2003).

Thanks to recent advances in neuroimaging, more and more structural asymmetries—in terms of cortical volume, surface area size, or thickness—are being discovered (for review, see Toga and Thompson, 2003). However, unlike the well-established relationship between planum temporale asymmetry and language dominance, whether those newly found structural asymmetries are related to the cognitive functions and to what extent remains largely unknown.

In a recent paper published in the Journal of Neuroscience, Plessen et al. (2014) set out to address these questions. Using high-resolution MRI, they measured brain asymmetry in terms of cortical thickness in a large sample (*n* = 215) with a wide age range (7–59 years old). Plessen et al. (2014) made three major findings regarding the relationship between brain asymmetry and cognitive function.

Firstly, they found that two asymmetries, a left > right asymmetry in posterior regions and a right > left asymmetry along the medial wall, are more pronounced in older adults. The left > right asymmetry in posterior regions, including inferior sensorimotor, inferior parietal, posterior temporal, and inferior occipital cortices, was primarily the result of the decrease in thickness with aging in the right hemisphere, and the increase in thickness with aging in the left hemisphere. This decrease in thickness with aging in posterior right hemisphere is consistent with the observation that tasks involving posterior right hemisphere are more susceptible to decline with aging (Gerhardstein et al., 1998). The increase in thickness with aging in posterior left hemisphere found by Plessen et al. (2014) is particularly enlightening. It revealed that the developmental thickness of the left-hemispheric posterior temporo-occipital region, first discovered in young children through to older adolescents (Shaw et al., 2009), may well persist into older adulthood. Moreover, the phenomenon that people generally gain more life knowledge, better emotion regulation and life satisfaction, become increasingly empathic and engage in more prosocial activities as we grow older, has long been acknowledged as aging wisdom (Charles and Carstensen, 2009). However, the specific structural basis remains unclear (Charles and Carstensen, 2009; Meeks and Jeste, 2009). Since the posterior areas of left hemisphere thickening with aging found by Plessen et al. (2014) are related to the attributes comprising wisdom (Meeks and Jeste, 2009), whether these areas are the structural substrate of aging wisdom deserves further study.

The right > left asymmetry along the medial wall, including the dorsal and posterior cingulate and the medial parietal and sensorimotor cortices, primarily resulted from the decrease in thickness with aging in the left hemisphere. This finding added greatly to our current understanding of brain aging loss. It suggested that the dominant right hemi-aging hypothesis that the right hemisphere shows greater age-related decline than the left is incomplete and needs to be revisited (Dolcos et al., 2002). However, as the participants in this study were comparatively young (≤59 years old), whether or not these findings would hold true for older people remains subject to further investigation.

Secondly, Plessen et al. (2014) found a significant gender difference, in which a left > right asymmetry occurred in females in anterior regions such as inferior temporal cortices, while a right > left asymmetry occurred in males in posterior regions, including orbitofrontal, inferior parietal, and inferior occipital cortices. As anterior regions are more involved in language abilities and posterior regions in visuospatial tasks, this may well explain the widely-known gender differences that males are more proficient at visuospatial tasks while females are more proficient at verbal tasks (Pease and Pease, 2000). However, the possible explanations for the first two findings by Plessen et al. (2014) mentioned above are mostly reasoned from previous observations. The authors themselves did not measure age or gender specific abilities and relate them to the asymmetry. In this regard, the third finding of Plessen et al. (2014) is invaluable.

Thirdly, Plessen et al. (2014) measured participants' working memory digit span and vocabulary performance using tests from the WAIS in adults or the “Number” subscale of the Children's Memory Scale and the WISC in children. Since both tasks are believed to be left-lateralizing, a left > right asymmetry correlating positively with the scores would be expected. Indeed, the authors found positive correlations between the digit span scores and the left > right asymmetries in the dorsolateral frontal and parietal cortices. As the extent of asymmetry increased, digit span score also increased. This is consistent with the left-lateralizing view of working memory.

However, Plessen et al. (2014) found inverse correlations between the digit span scores and the left-right asymmetries along the medial walls, including the medial posterior parietal cortex, dorsolateral portions of the frontal and parietal cortices. They also found inverse correlations between the vocabulary scores and the left-right asymmetries along the lateral surface of the temporal lobe, the posterior medial wall, and the inferior surface of the brain. A right > left asymmetry in these regions is therefore positively correlated with digit span and vocabulary score. Coincidentally, the medial posterior parietal cortex has been implicated in working memory tasks (Owen et al., 2005). The right anterior temporal lobe, right superior temporal gyrus, and right inferior frontal gyrus are also activated during inference and semantic processing (Virtue et al., 2008; Kounios and Jung-Beeman, 2009). These findings challenge the view that working memory and vocabulary tasks are left-lateralized.

Nevertheless, this third finding by Plessen et al. (2014) is consistent with previous reports that have shown that, whereas patients with developmental dyslexia show decreased left > right asymmetry in planum temporale, musicians especially those with perfect pitch show exaggerated left > right asymmetry in planum temporale (see Toga and Thompson, 2003). As expected, the planum temporale is involved in language-related auditory processing and music perception.

In summary, the combined findings provide compelling evidence for the correlation between structural asymmetry and cognitive function, supporting the “bigger is better” structure-function relationship. They also raise an interesting question regarding the underlying causality. Is structural asymmetry the basis or prerequisite of the function, or only the result of functional training? This naturally brings to mind the age-old debate of nature vs. nurture. Encouragingly, several longitudinal studies on musical training have provided promising evidence supporting experience induced brain plasticity, or “nurture” (Herholz and Zatorre, 2012). Following acquisition of musical skills through extensive training, brain structures supporting these skills change and predict performance. More studies on the effect of cognitive training on brain asymmetry as well as its cellular and molecular mechanism are needed to further our understanding of this inquiry.

## Conflict of interest statement

The authors declare that the research was conducted in the absence of any commercial or financial relationships that could be construed as a potential conflict of interest.
